# Chemistry of Materials for Energy and Environmental Sustainability

**DOI:** 10.3390/molecules29245929

**Published:** 2024-12-16

**Authors:** Qinguo Shao

**Affiliations:** 1 School of Intelligent Manufacturing and Control Engineering, Shandong Institute of Petroleum and Chemical Technology, Dongying 257061, China; qgshao@upc.edu.cn; 2 Dongying Key Laboratory of Mechanical Surface Engineering and Corrosion Protection, Shandong Institute of Petroleum and Chemical Technology, Dongying 257061, China; 3 Shandong Provincial Engineering Research Center for Green Manufacturing and Intelligent Control, Shandong Institute of Petroleum and Chemical Technology, Dongying 257061, China

In contemporary society, energy serves as the cornerstone of human survival and development, exerting a profound influence on the economic development of nations and the trajectory of global progress. On one hand, the gradual depletion of non-renewable resources coupled with the rapid escalation of energy demand poses significant challenges [1,2,3]. On the other hand, the combustion of fossil fuels such as coal and petroleum leads to atmospheric pollution and the emission of toxic gasses, including sulfides, nitrides, and carbon dioxide contributing to the greenhouse effect, all of which have severely impacted the global climate and environment. Currently, air pollution has emerged as a pressing global issue. Consequently, research and development regarding clean energy sources play a pivotal role in ensuring the sustainable development of national economies [4,5,6,7,8]. Efforts are continuously being made to explore various renewable energy sources, including wind, solar, hydro, and tidal power, and notable progress has been achieved in this domain [9,10,11,12,13,14,15]. However, these renewable energy sources, which rely on natural conditions, inherently possess certain drawbacks. These include the intermittency of power generation, discontinuity in generation periods, and the uncontrollability of generation intensity, resulting in an unstable electrical output. Therefore, there is a crucial need for energy storage devices that can rapidly store this intermittent and unstable clean energy, thereby enabling the establishment of a continuous and stable energy supply system through these storage solutions. In all kinds of energy storage systems, electric energy storage systems occupy a key position, and batteries and electrochemical capacitors have become indispensable energy storage devices [16,17,18,19]. Advanced electrode materials are the key components of electrochemical energy systems. Strategies for increasing their energy densities include tailoring the chemistry, structure, and components of the electrode materials [20,21,22,23,24,25,26]. Although tremendous effort has been devoted to this field of research, revolutionary energy storage devices with both high energy densities and power densities still remain a challenge [27,28,29,30,31,32,33].

At the same time, with the rapid growth of the global population and the accelerated development of industry, freshwater resources are becoming increasingly scarce and severely polluted [34,35,36,37,38,39,40,41]. It is reported that by 2030, the world will face a shortage of potable water resources, and concerns over the safety of drinking water have also gained widespread attention. Approximately 3.1 million people die annually from diseases caused by unsafe drinking water. Water resource issues impact food production, industrial output, and environmental quality, further affecting the industrialized economic development of countries [42,43,44,45,46,47,48,49,50,51,52,53]. Therefore, the effective treatment of water pollution, the desalination of seawater resources, and the purification of drinking water resources are crucial means to address the current challenges [54,55,56,57,58,59,60,61,62,63,64,65,66,67,68].

The aim of this Special Issue is to publish original research articles and review papers on chemistry research regarding advanced materials relevant to energy and environmental sustainability. This Special Issue contains twelve papers, including one comprehensive review and eleven research papers. The review paper by Chen et al. [69] discusses carbon dots derived from non-biomass waste. The authors introduce various preparation methods, diverse applications, and future challenges of carbon dots. In this Special Issue, several papers focus on energy storage and conversion materials. Liu et al. [70] develop a high-performance dual-ion battery using a silicon–graphene composite as the anode and expanded graphite as the cathode. In this design, the stress/strain induced by electrode volume change during charging and discharging can be suppressed and the obtained full Si@G//EG DIBs exhibit a high energy density of 367.84 Wh kg^−1^ at a power density of 855.43 W kg^−1^. This work sheds some light on the practical applications of high-energy DIBs. Manganese molybdate has been regarded as a promising electrode material for supercapacitors. However, its low electrical conductivity mainly blocks its application in practice. Liu and Li et al. [71] synthesize phosphorus-doped MnMoO_4_·H_2_O nanosheets by means of a hydrothermal method and use them as an electrode material in an asymmetric supercapacitor. Owing to the phosphorus–metal bonds and oxygen vacancies induced by phosphorus element doping, the charge storage and conductivity of the electrode are increased, thereby resulting in enhanced electrochemical properties with a high energy density of 41.9 Wh kg^−1^ under 666.8 W kg^−1^. Xie et al. [72] study the effect of [BMP]^+^ [BF4]^−^ additives on the performance of CsPbI_1.2_Br_1.8_ solar cells. It is found that the additive could effectively reduce the phase segregation phenomenon of the CsPbI_1.2_Br_1.8_ films. Inerbaev et al. [73] present a calculation result demonstrating that doping BaTiO_3_ with Rh could reduce the overpotential of oxygen evolution when it was used as a catalyst. Rh doping could expand the spectrum of absorbed light to the entire visible range. Gaur et al. [74] report using hen feathers as an adsorbent in aqueous media. Their results show that hen feathers exhibit high impressive adsorption efficiency towards Metanil Yellow dye. Liu et al. [75] develop a high-performance nanofiltration membrane which exhibits a rejection rate of over 99% for various dyes. The nanofiltration is prepared using TEMPO-oxidized cellulose nanofibers via the vacuum filtration method. Yang et al. [76] report the use of activated red mud particles as adsorbents for phosphorus adsorption and the possible mechanism is examined by means of morphology analysis, FTIR, EDS, and mineral composition analysis. Kuppadakkath et al. [77] demonstrate that modified BTA molecules show anion-responsive properties and that they can also be used as adsorbents for hazardous dyes in water. Ni et al. [78] reveal that the mechanism of degradation of the photosensitive pollutant tetracycline is promoted by GO, which offers reference value for research in wastewater treatment. Wang et al. [79] prepare a new zwitterionic polymer and use it to construct microfiltration membranes. Their experiment showed that the as-prepared membranes exhibit excellent efficiency in oil–water separation. Fu et al. [80] prepare a cobalt–iron bimetallic modified hydrogen-type mordenite by means of the ion exchange method, which shows superior DME carbonylation catalytic activity and stability. In addition, the possible reasons for the improvement are clarified.

In summary, this Special Issue presents the latest research on the chemistry of materials for energy and environmental sustainability. From rationally designed composite electrode materials for energy storage and effective additives for promoting solar cells to powerful adsorbents of hazardous dyes in water and versatile membranes for oil–water separation, these reports showcase the state-of-the art material tailoring in the energy and environmental sustainability field. The advances in this Special Issue may shed some new light on possible solutions to energy and environmental challenges.
